# European Subtype Tick-borne Encephalitis Virus in *Ixodes persulcatus* Ticks

**DOI:** 10.3201/eid1702.101487

**Published:** 2011-02

**Authors:** Anu E. Jääskeläinen, Elina Tonteri, Tarja Sironen, Laura Pakarinen, Antti Vaheri, Olli Vapalahti

**Affiliations:** Author affiliations: University of Helsinki, Helsinki, Finland (A.E. Jääskeläinen, E. Tonteri, T. Sironen, A. Vaheri, O. Vapalahti); National Institute for Health and Welfare, Helsinki (L. Pakarinen);; Hospital District of Helsinki and Uusimaa, Helsinki (A. Vaheri, O. Vapalahti)

**Keywords:** vector-borne infections, tick-borne encephalitis virus, viruses, TBEV, Ixodes, Finnish Lapland, letter

**To the Editor:** The northernmost tick-borne encephalitis (TBE) focus is in Simo, Finnish Lapland. Four TBE cases were confirmed during 2008–2009. Tick-borne encephalitis virus (TBEV) is transmitted by *Ixodes* spp. ticks and is endemic to Eurasia from central Europe to the Far East. The virus has 3 subtypes: European (TBEV-Eur), Siberian (TBEV-Sib), and Far Eastern (TBEV-FE). TBEV-Eur is mainly transmitted by *I. ricinus* ticks (sheep ticks) and the 2 other subtypes by *I. persulcatus* ticks (taiga ticks). The range of *I. ricinus* ticks covers most of continental Europe and the British Isles; *I. persulcatus* ticks are distributed throughout eastern Europe and Asia to the People’s Republic of China and Japan.

The transmission cycle of at least TBEV-Eur in nature is fragile and depends on microclimatic conditions. Thus, within the *I. ricinus* distribution area, TBE is endemic merely focally (*1*,*2*). In Finland, TBE foci are located by the sea or large lakes (Figure A1). Both vector tick species are found: *I. ricinus* ticks in the southern and central parts of the country, but *I. persulcatus* ticks are in scattered foci along the western coast, including the Kokkola archipelago and Närpiö municipality, where they carry TBEV-Sib (*3*,*4*) (Figure A1).

The first human TBE cases from Simo in Lapland (65°40′N, 24°54′E; Figure A1) were reported during 2008 (n = 2) and 2009 (n = 2). On the basis of interviews with the 2 patients from 2008, we collected 97 ticks and 17 bank voles from the 2 probable sites of infection during June 2009. From the rodents, we extracted blood from the heart and performed TBEV-antibody tests by immunofluorescence assay. The ticks were placed in 51 pools (1–3 ticks/pool). We isolated RNA from tick pools and rodent lungs and brains by TriPure Isolation Reagent (Roche Diagnostics, Indianapolis, IN, USA) and performed real-time reverse transcription–PCR (*5*) to detect TBEV RNA. For the positive tick pools, we confirmed the identification species by *Ixodes* mtDNA sequencing (*6*).

Six of 51 tick pools (with a total of 97 *I. persulcatus* ticks) were positive for TBEV in real-time reverse transcription–PCR, resulting in 6% TBEV RNA prevalence. At least 1 organ was positive for TBEV RNA in as many as 15/17 bank voles, in line with our finding that TBEV RNA persists in rodents for months (*7*); 4 rodents had antibodies to TBEV. The TBEV RNA prevalence among ticks and rodents was relatively high, as is the incidence among humans (0.57 cases/year/1,000 inhabitants) in Simo, indicating a focus with high activity.

We isolated 6 TBEV strains from suckling mice (experimental animal permit ESLH-2008–06558/Ym-23): 2 from *I. persulcatus* tick pools (Simo-38 and Simo-48; pools of 2 and 3 ticks, respectively), and 4 from TBEV antibody– and RNA-positive rodent lung–brain suspensions (Simo-2, -5, -7 and -9). Partial envelope (E) and nonstructural protein 3 genes (*4*) of the isolated TBEV strains were sequenced (accession nos. HQ228014–HQ228024, GenBank) and subjected to phylogenetic analysis (Figure A1). Within the 1208 nt from the E gene, Simo-38 and Simo-48 from ticks and Simo-9 from a bank vole were identical. Other sequences differed for 1 nt and Simo-2 for 1 aa compared with the others. All strains were monophyletic and belonged to the TBEV-Eur subtype. The partial nonstructural protein 3 gene sequences were identical, and the phylogenetic tree showed similar topography as for the E gene (not shown).

The only tick species found in Simo was *I. persulcatus,* further widening its known distribution along the western coast of Finland (Figure A1). However, the virus subtype found in Simo was TBEV-Eur strain, the main vector of which is the *I. ricinus* tick*.*

TBEV-Eur strains are commonly very closely related to each other and do not form clear geographic clusters (*4*). Thus, it is difficult to deduce the origin of the virus. The nearest TBE-endemic focus is the Kokkola archipelago, ≈200 km south (Figure A1), but there *I. persulcatus* ticks carry the TBEV-Sib strain (*3*). The nearest areas to which the TBEV-Eur strain is endemic are in southern Finland where only *I. ricinus* ticks have been found.

Cattle serum samples were negative for antibodies to TBEV in the Simo area in the 1960s (*8*). The first human TBE cases from Simo were identified during 2008 and 2009. We isolated TBEV strains from ticks and rodents in 2009. Simo appears to be a recently established, and the northernmost, TBE focus known. TBEV may have been introduced to Simo from a geographically distinct location recently, likely within the past 50 years.

TBE seems to be moving northward in Europe (*9*) and shifting upward to higher elevations in the mountains (*10*), apparently influenced by climate change. An altered microclimate favoring TBE circulation (*1*), in addition to introduction of the virus, could also explain the recent emergence of TBE in Simo. In conclusion, Simo in Finnish Lapland is a new TBE-endemic focus demonstrating northward movement of foci and an unusual combination of the TBEV-Eur strain and *I. persulcatus* ticks in an area with no evidence of cocirculation of tick species or TBEV subtypes.

## Figures and Tables

**Figure A1:**
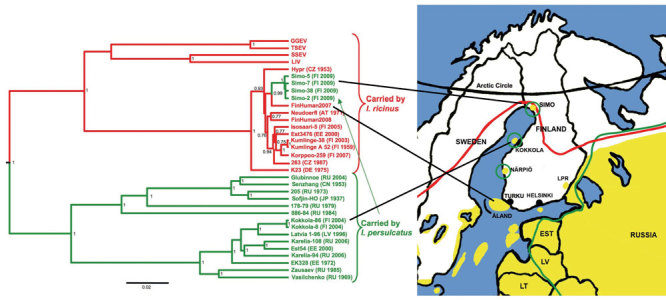
A1. Phylogenetic tree of partial E (1172-nt) gene sequences, shwoing Ixodes ricinus–transmitted strains (red) and I. persulcatus–transmitted strains (green). The tree was reconstructed by the Bayesian Markov chain Monte Carlo method in BEAST (http://beast.bio.ed.ac.uk). Maximum clade credibility tree with an arbitrary root is shown with mean branch lengths, and Bayesian posterior probabilities are given at nodes when >0.7. Country of origin and isolation year are indicated. Four strains from Simo are shown; Simo-48 and Simo-9 were identical to Simo-38. Yellow shading, tick-borne encephalitis–endemic areas; red line, I. ricinus distribution; green line, I. persulcatus distribution; LPR, Lappeenranta. Scale bar indicates number of substitutions per site.

## References

[1] Randolph SE, Green RM, Peacey MF, Rogers DJ. Seasonal synchrony: the key to tick-borne encephalitis foci identified by satellite data. Parasitology. 2000;121:15–23. 10.1017/S0031182099006083 11085221

[2] Lindquist L, Vapalahti O. Tick-borne encephalitis. Lancet. 2008;371:1861–71. 10.1016/S0140-6736(08)60800-4 18514730

[3] Jääskeläinen AE, Tikkakoski T, Uzcátegui NY, Alekseev AN, Vaheri A, Vapalahti O. Siberian subtype tickborne encephalitis virus, Finland. Emerg Infect Dis. 2006;12:1568–71. 17176574 10.3201/eid1210.060320 PMC3290944

[4] Jääskeläinen AE, Sironen T, Murueva GB, Subbotina N, Alekseev AN, Castrén J, Tick-borne encephalitis virus in ticks in Finland, Russian Karelia, and Buryatia. J Gen Virol. 2010;91:2706–12. 10.1099/vir.0.023663-0 20660147

[5] Schwaiger M, Cassinotti P. Development of a quantitative real-time RT-PCR assay with internal control for the laboratory detection of tick borne encephalitis virus (TBEV) RNA. J Clin Virol. 2003;27:136–45. 10.1016/S1386-6532(02)00168-3 12829035

[6] Caporale DA, Rich SM, Spielman A, Telford SR III, Kocher TD. Discriminating between *Ixodes* ticks by means of mitochondrial DNA sequences. Mol Phylogenet Evol. 1995;4:361–5. 10.1006/mpev.1995.1033 8747292

[7] Tonteri E, Jääskeläinen AE, Tikkakoski T, Voutilainen L, Niemimaa J, Henttonen H, Tick-borne encephalitis virus in wild rodents in winter, Finland, 2008–2009. Emerg Infect Dis. 2011;17:72–5. 10.3201/eid1701.100051 21192857 PMC3204619

[8] Tuomi J, Brummer-Korvenkontio M. Antibodies against viruses of the tick-borne encephalitis group in cattle sera in Finland. Ann Med Exp Biol Fenn. 1965;43:149–54. 5893666

[9] Randolph SE, Rogers DJ. Fragile transmission cycles of tick-borne encephalitis virus may be disrupted by predicted climate change. Proc Biol Sci. 2000;267:1741–4. 10.1098/rspb.2000.1204 12233771 PMC1690733

[10] Lukan M, Bullova E, Petko B. Climate warming and tick-borne encephalitis, Slovakia. Emerg Infect Dis. 2010;16:524–6. 10.3201/eid1603.081364 20202437 PMC3321998

